# Epstein-Barr virus encoded latent membrane protein 1 suppresses necroptosis through targeting RIPK1/3 ubiquitination

**DOI:** 10.1038/s41419-017-0081-9

**Published:** 2018-01-19

**Authors:** Xiaolan Liu, Yueshuo Li, Songling Peng, Xinfang Yu, Wei Li, Feng Shi, Xiangjian Luo, Min Tang, Zheqiong Tan, A. M. Bode, Ya Cao

**Affiliations:** 1 Key Laboratory of Carcinogenesis and Invasion, Chinese Ministry of Education, Xiangya Hospital, Central South University, Changsha, Hunan China; 2 Cancer Research Institute, Xiangya School of Medicine, Central South University, Changsha, Hunan China; 3 Key Laboratory of Carcinogenesis, Chinese Ministry of Health, Changsha, Hunan China; 4 The Hormel Institute, University of Minnesota, Austin, MN USA

## Abstract

Necroptosis is an alternative programmed cell death pathway that is unleashed in the absence of apoptosis and mediated by signaling complexes containing receptor-interating protein kinase 1 (RIPK1) and RIPK3. This form of cell death has recently been implicated in host defense system to eliminate pathogen-infected cells. However, only a few viral species such as herpes simplex virus (HSV) and cytomegalovirus (CMV) have evolved mechanisms inhibiting necroptosis to overcome host antiviral defense, which is important for successful pathogenesis. Here, we show that the γ-herpesvirus Epstein–Barr virus (EBV) blocks necroptosis in EBV-infected human nasopharyngeal epithelial cells and nasopharyngeal carcinoma cells. Our findings indicate that EBV-encoded latent membrane protein 1 (LMP1), which lacks an RIP homotypic interaction motif (RHIM) domain, has mechanisms distinct from RHIM signaling competition to inhibit this necroptotic pathway. Intriguingly, LMP1 interacts directly with both RIPK1 and RIPK3 through its C-terminal activation region. More importantly, LMP1 can modulate the post-translational modification of the two receptor-interacting proteins. We then show that LMP1-mediated promotion of K63-polyubiquitinated RIPK1, suppression of RIPK1 protein expression and inhibition of K63-polyubiquitinated RIPK3 induced a switch in cell fate from necroptotic death to survival. These findings provide direct evidence for the suppression of necroptosis by EBV and define a mechanism of LMP1 to interrupt the initiation process of necroptosis before necrosome formation.

## Introduction

Programmed necrosis or necroptosis has emerged as a novel form of programmed cell death that is independent of caspase activity. To date, the best characterized necroptosis pathway is triggered by tumor necrosis factor α (TNFα), which requires the receptor-interating protein kinase 1 (RIPK1) and RIPK3^1^. In TNFα-induced necroptosis, RIPK1 and RIPK3 form a protein complex termed the ‘necrosome’ through their respective RIP homotypic interaction motif (RHIM) domains^2^. Necrosome formation leads to the activation and phosphorylation of RIPK3. The phosphorylation at Ser227 is required for human RIPK3 to recruit and phosphorylate downstream substrate protein mixed lineage kinase domain-like (MLKL)^3^. Upon phosphorylation, MLKL forms an oligomer that moves from the cytosol to the plasma and intracellular membranes^4^, thereby disrupting membrane integrity and resulting in necrotic death. In addition to RIPK1 and RIPK3, the RHIM is also found in TRIF^5^, DAI/ZBP1^6^, and ICP6^7,8^, which can interact with RIPK3 to form RHIM-dependent signaling complexes.

Like apoptosis, necroptosis has recently been implicated in eliminating pathogen-infected cells as a component of host defense against infection. This contribution was derived for the first time from studies of vaccinia virus, which facilitates TNFα-induced necroptosis in human and mouse cells^9,10^. Additionally, a number of investigations demonstrated that some viruses could balance the host defense against infection. In the case of MCMV infection, this virus encodes a RHIM-containing protein, a viral inhibitor of RIP activation (vIRA/M45), and thus inhibits necroptosis in mouse cells by disrupting RHIM-dependent signal transduction^11^. Another member of the β-herpesvirus subfamily, human cytomegalovirus, has also been shown to block necroptotic cell death in human fibroblasts^12^. Furthermore, the α-herpesvirus subfamily members HSV-1 and HSV-2 display opposing activities in manipulating necroptosis depending upon the host species. In human cells, the HSV-1 ribonucleotide reductase large subunit ICP6 (ICP10 for HSV-2) prevents TNFα-induced necroptosis by inhibiting the interaction between RIPK1 and RIPK3 through its N-terminal RHIM domain and by blocking the activity of caspase-8 through its C-terminal caspase-8 binding domain^13^. In contrast to its behavior in human cells, HSV activates necroptosis in mouse cells and mice^7,8^. The outcome of HSV infection likely depends on the species-specific evolutionary development between the virus and its host.

Notably, all four of the necroptosis inhibitory viruses belong to herpesvirus family and commonly establish a life-long infection of the host. As such, viral manipulation of the host cell death signaling pathway provides these pathogens with the opportunity to maintain infection and facilitate viral replication. Previous work from our laboratory and others suggested that the Epstein–Barr virus (EBV), a human γ-herpesvirus, has evolved multiple mechanisms to inhibit host cell apoptosis following infection^14–17^. Like other herpesviruses, the life cycle of EBV consists of latent and lytic replication phases. The EBV early lytic cycle protein BHRF1, containing regions with extensive homology to Bcl-2, is capable of protecting cells from apoptotic cell death^14^. Another EBV product, latent membrane protein 1 (LMP1) has also been shown to prevent apoptosis by upregulating the expression of anti-apoptotic molecules^15,16^. Although the control of apoptosis by EBV products has been well characterized, the relationship between EBV and host cell necroptosis remains undefined.

In this study, we found that EBV prevents necroptosis in human nasopharyngeal epithelial cells and nasopharyngeal carcinoma cells. Further mechanistic studies revealed that the suppression was mainly due to the EBV-encoded LMP1. Interestingly, although LMP1 lacks the RHIM domain, it is able to interact with both RIPK1 and RIPK3 through its C-terminal activation region. Moreover, LMP1 promotes K63-linked polyubiquitination of RIPK1 and suppresses the protein expression while inhibiting K63-linked polyubiquitination of RIPK3, which contributes to the activation of NF-κB and disruption of necrosome formation, collectively switching cell fate from death to survival. Our findings provide evidence supporting an important role of EBV-LMP1 in resisting host cell necroptosis and enrich our knowledge regarding the pathogens that could subvert and evade this kind of host defense.

## Results

### EBV inhibits host cell necroptosis

**Fig. 1:**
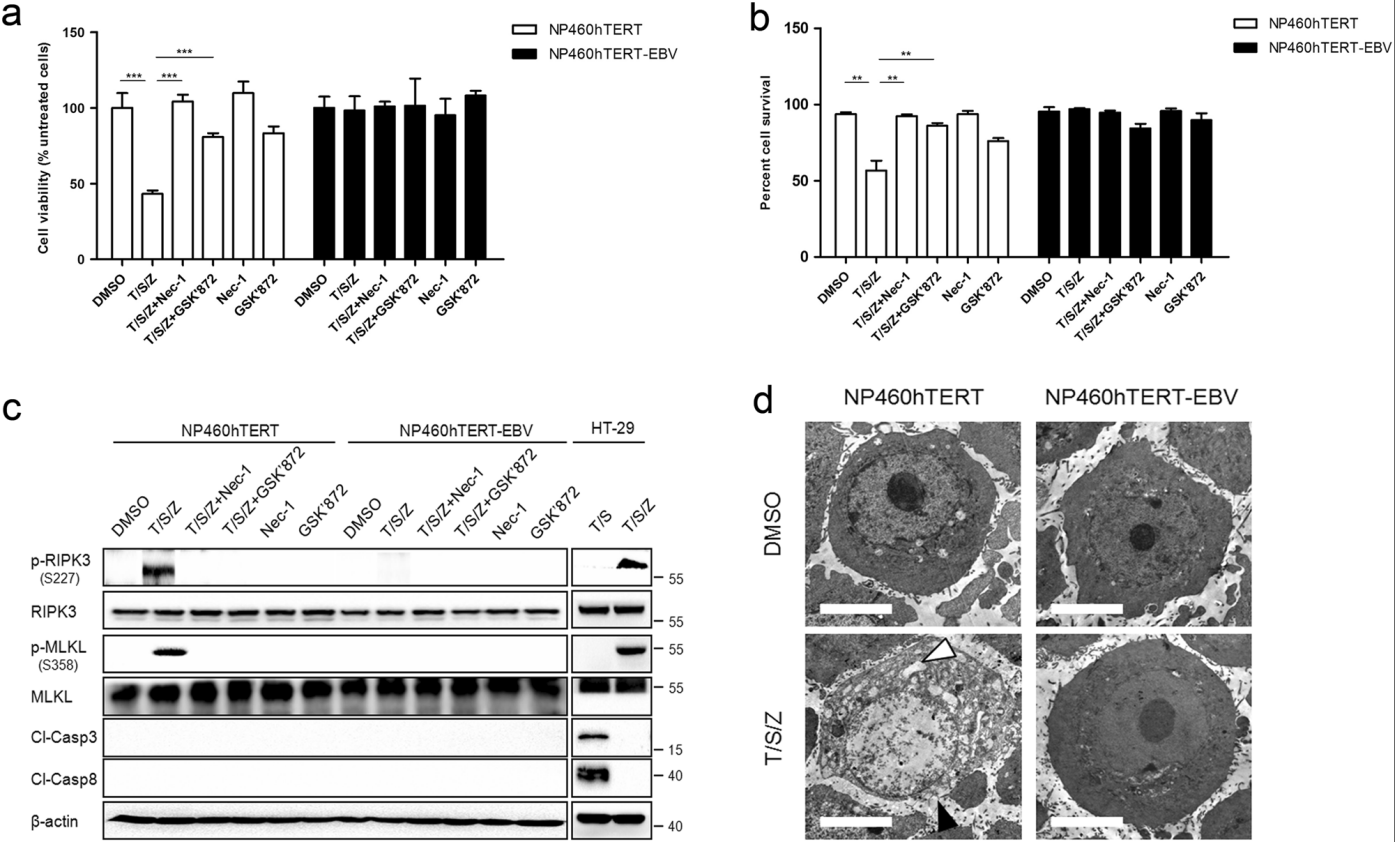
**EBV infection prevents T/S/Z-induced necroptosis.** **a**,** b** NP460hTERT and NP460hTERT-EBV cells were treated with T/S/Z, T/S/Z + Nec-1, T/S/Z + GSK’872, Nec-1, GSK’872, or DMSO solvent control for 24 h. At the end of treatment, cell viability and cell survival were determined by MTS and trypan blue exclusion assay. Data were presented as mean ± S.E.M. (*n* = 3). *P* values are by Student’s *t* test. T TNF-α, 100ng/ml; S Smac mimetic, 5 μM; Z z-VAD-fmk, 20 μM; Nec-1 Necrostatin-1, 40 μM; GSK’872: 5 μM. **c** Immunoblot analysis to detect phosphorylated RIPK3 (p-RIPK3), RIPK3, phosphorylated MLKL (p-MLKL), MLKL, cleaved Caspase-3 (Cl-Casp3), cleaved Caspase-8 (Cl-Casp8), and β-actin in lysates from NP460hTERT and NP460hTERT-EBV cells treated with T/S/Z, T/S/Z + Nec-1, T/S/Z + GSK’872, Nec-1, GSK’872, or DMSO solvent control and HT-29 cells treated with T/S or T/S/Z as indicated. **d** TEM photomicrographs of NP460hTERT and NP460hTERT-EBV cells treated with DMSO or T/S/Z. Black and white arrows denote plasma membrane rupture and cellular organelle swelling, respectively. Scale bars represent 5 μm

A variety of cell lines have been shown to undergo necroptosis in response to T/S/Z^18–20^. This kind of programmed cell death can be pharmacologically inhibited by chemical compounds such as necrostatin-1^21^ or GSK’840/843/872^22^. In order to investigate the effect of EBV on host cell necroptosis, we treated EBV infected and uninfected human nasopharyngeal epithelial cells with necroptosis inducers (T/S/Z) and/or the inhibitor Nec-1 or GSK’872. Our results showed that T/S/Z induction led to massive cell death of NP460hTERT cells and this pattern of cell death was prevented to a certain extent by the necroptosis specific inhibitor Nec-1 or GSK’872 (Fig. 1a, b), indicating that NP460hTERT cells were sensitive to T/S/Z-induced necroptosis. However, the survival rate of NP460hTERT-EBV cells was unaltered after T/S/Z treatment. As RIPK3 phosphorylation at S227 and MLKL phosphorylation at T357/S358 is critical for the induction of necroptosis, we then performed an immunoblot analysis of p-RIPK3 and p-MLKL as well as markers of apoptosis including cleaved caspase-3 and cleaved caspase-8 to confirm that the type of cell death is necroptosis rather than apoptosis. Meanwhile, T/S and T/S/Z treated HT-29 were used as control apoptotic and necroptotic cells^23^ in the immunoblot assay. Consistent with the cell viability assay results, T/S/Z-induced excessive expression of p-RIPK3 and p-MLKL in NP460hTERT cells, which was significantly suppressed by treatment with Nec-1 or GSK’872 (Fig. 1c). In contrast, p-RIPK3 and p-MLKL was hardly observed in NP460hTERT-EBV cells following T/S/Z treatment. Subsequently, ultrastructural features of NP460hTERT and NP460 hTERT-EBV cells were analyzed by transmission electron microscopy (TEM). While most of the DMSO treated NP460hTERT cells showed normal cellular morphology, a significant percentage of the cells displayed necrotic morphology in response to T/S/Z, including numerous swollen cellular organelles as well as discontinuous cytoplasmic membrane (Fig. 1d; Supplementary Table 1). On the other hand, no morphological difference was observed between DMSO and T/S/Z-treated NP460hTERT-EBV cells. Taken together, these data indicate that EBV inhibits T/S/Z-induced necroptosis.

### EBV-LMP1 contributes to the suppression of T/S/Z-induced necroptosis

**Fig. 2:**
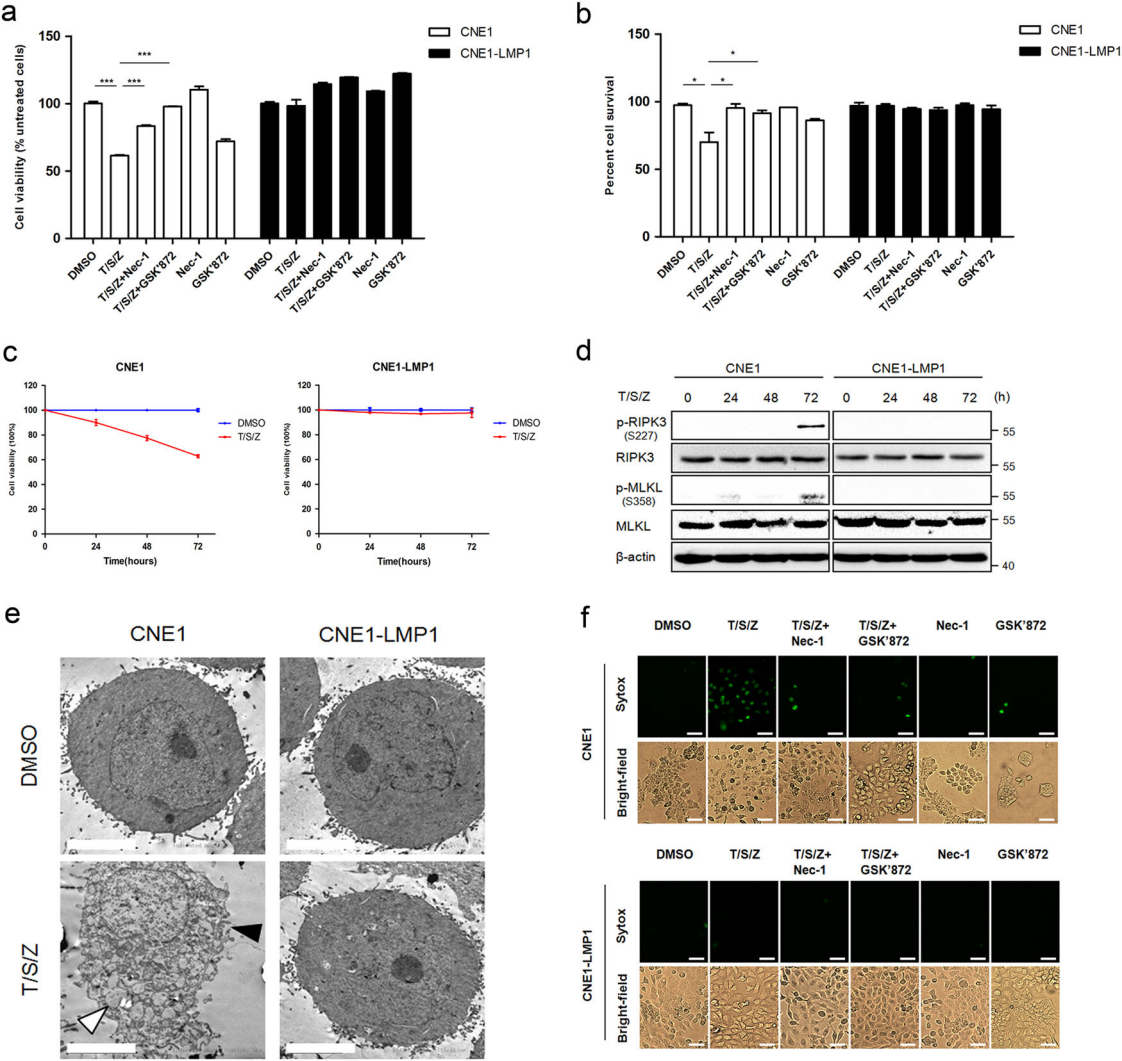
**EBV-LMP1 plays a role in suppression of T/S/Z-induced necroptosis.** **a**, **b** CNE1 and CNE1-LMP1 cells were treated with T/S/Z, T/S/Z + Nec-1, T/S/Z + GSK’872, Nec-1, GSK’872, or DMSO solvent control for 72 h. At the end of treatment, cell viability, and cell survival were determined by MTS and trypan blue exclusion assay. Data were presented as mean ± S.E.M. (*n* = 3). *P* values are by Student’s *t* test. T TNF-α, 100ng/ml; S Smac mimetic, 5 μM; Z z-VAD-fmk, 20 μM; Nec-1 Necrostatin-1, 40 μM; GSK’872: 5 μM. **c** CNE1 and CNE1-LMP1 cells were treated with T/S/Z or DMSO solvent control for 0, 24, 48, or 72 h. At the end of treatment, cell viability was determined by MTS assay. **d** Immunoblot analysis to detect phosphorylated RIPK3 (p-RIPK3), RIPK3, phosphorylated MLKL (p-MLKL), MLKL, and β-actin in lysates from CNE1 and CNE1-LMP1 cells treated with T/S/Z for 0, 24, 48, or 72 h. **e** TEM photomicrographs of CNE1 and CNE1-LMP1 cells treated with DMSO or T/S/Z. Black and white arrows denote plasma membrane rupture and cellular organelle swelling, respectively. Scale bars represent 5 μm. **f** CNE1 and CNE1-LMP1 cells were treated with T/S/Z, T/S/Z + Nec-1, T/S/Z + GSK’872, Nec-1, GSK’872, or DMSO solvent control for 72 h. Cells were then stained with Sytox Green and imaged by a fluorescent microscope. Scale bars represent 50 μm

Next, we focused on the ability of EBV-encoded latent protein LMP1 to inhibit T/S/Z-induced necroptosis. LMP1-overexpressing narsopharyngeal carcinoma cells (CNE1-LMP1) as well as the parental cells (CNE1) were used first to assess LMP1 function. Cell viability assay results showed that T/S/Z-induced moderate cell death in CNE1 cells (Fig. 2a, b). In addition, immunoblot analysis of cleaved caspase-3 and cleaved caspase-8 confirmed that the type of cell death was not apoptosis (Supplementary Fig. 1). In contrast, CNE1-LMP1 cells resisted cell death induced by T/S/Z. Unlike NP460hTERT, CNE1 cells required 72 h of T/S/Z treatment to induce the expression of p-PIPK3 and sufficient p-MLKL to execute necroptosis (Fig. 2c, d), while p-PIPK3 and p-MLKL was hardly observed in CNE1-LMP1 cells at all the time points examined. Subsequently, TEM analysis revealed that although some of the CNE1 cells underwent necrotic-like cell death following T/S/Z treatment, no morphological changes were observed in CNE1-LMP1 cells (Fig. 2e; Supplementary Table 2). Since necroptosis is characterized by plasma membrane rupture^1^, we used Sytox Green fluorescence staining to detect the integrity of cellular membrane. Our results showed that CNE1-LMP1 cells remained impermeable to Sytox Green uptake following T/S/Z induction, which was in stark contrast to the pattern of necroptosis with increased Sytox Green fluorescence in CNE1 cells (Fig. 2f). The importance of LMP1 in inhibiting necroptosis is further confirmed by the observation that knockdown of LMP1-sensitized EBV-positive nasopharyngeal carcinoma cell line C666-1 to T/S/Z-induced cell death (Supplementary Fig. 2). Summing up the above data, it can be demonstrated that EBV-LMP1 contributes to the viral suppression of T/S/Z-induced necroptosis in narsopharyngeal carcinoma cells.

### LMP1 interacts directly with RIPK1 and RIPK3

**Fig. 3:**
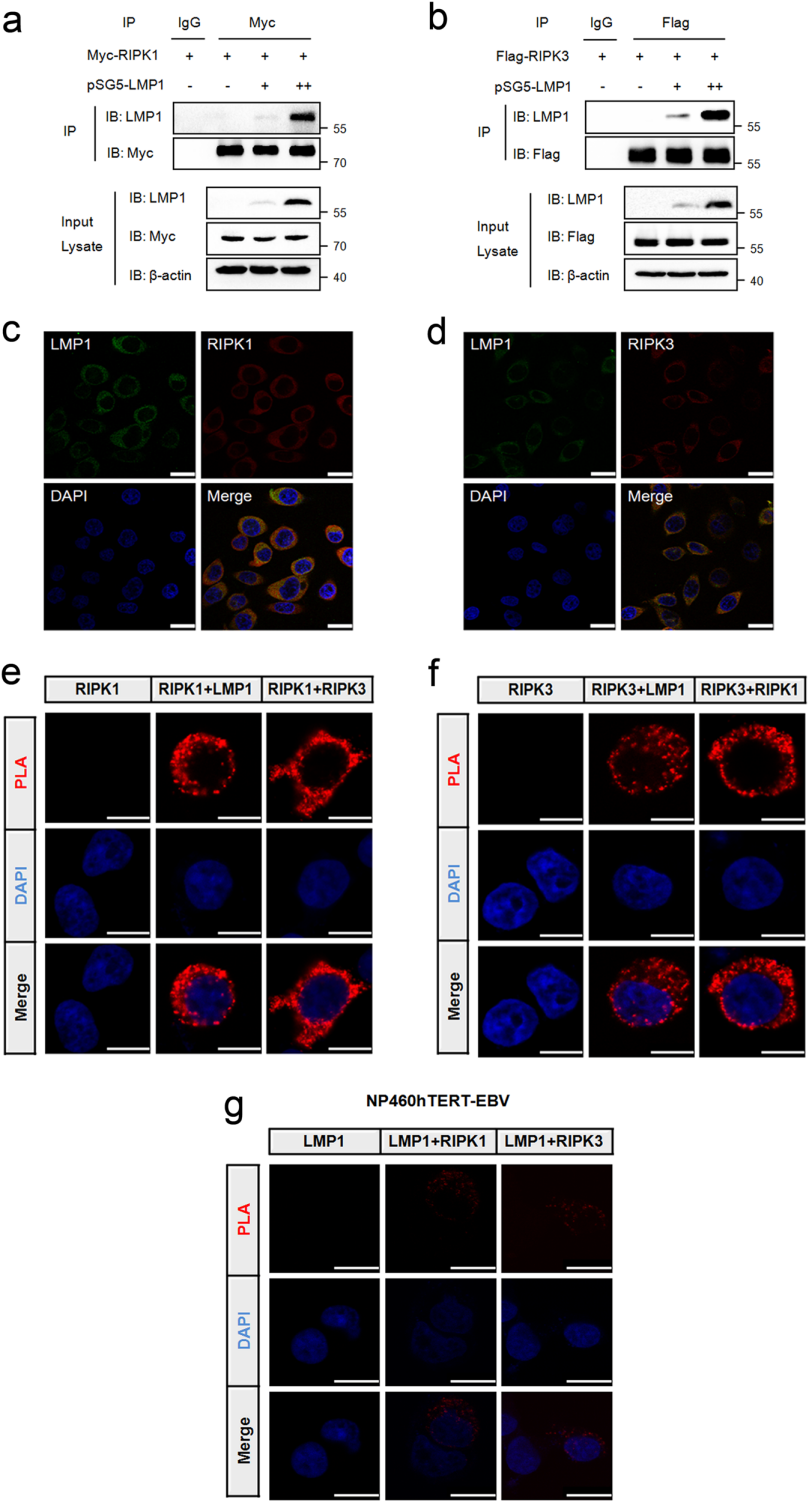
**LMP1 interacts directly with RIPK1 and RIPK3.** **a** IP/IB was used to detect the interaction of LMP1 with RIPK1. Top panels show lysates of 293T cells transfected with Myc-RIPK1 and different amounts of pSG5-LMP1 subjected to IP with IgG or anti-Myc antibody followed by IB with anti-LMP1 or anti-Myc antibody. The lower panels depict IB of input cell lysates. Protein molecular size is shown to the right of the lanes. **b** IP/IB was used to detect the interaction of LMP1 with RIPK3. Top panels show lysates of 293T cells transfected with Flag-RIPK3 and different amounts of pSG5-LMP1 subjected to IP with IgG or anti-Flag antibody followed by IB with anti-LMP1 or anti-Flag antibody. The lower panels depict IB of input cell lysates. **c**, **d** CNE1-LMP1 cells were immunostained with anti-LMP1 (green) and anti-RIPK1 (red) or anti-RIPK3 (red) antibodies, and subjected to confocal microscopy. The nuclei were stained with DAPI. Scale bars represent 25 μm. **e**, **f** Proximity ligation assay was used to detect the LMP1–RIPK1 and LMP1–RIPK3 interactions in 293T cells transfected with the indicated expression plasmids (top of each panel). Red fluorescence corresponds to the PLA positive signal and blue fluorescence corresponds to nuclei (DAPI staining). Scale bars represent 10 μm. **g** Proximity ligation assay was used to detect the endogenous LMP1–RIPK1 and LMP–RIPK3 interactions in NP460hTERT-EBV cells treated with 10 μM MG132 for 16 h. Red fluorescence corresponds to the PLA positive signal and blue fluorescence corresponds to nuclei (DAPI staining). Scale bars represent 10 μm

To examine whether LMP1 could interact with the two receptor-interacting proteins, we co-expressed Myc-RIPK1 or Flag-RIPK3 together with increasing amounts of pSG5-LMP1 in 293T cells. Our result revealed that LMP1 interacted in a dose-dependent manner with exogenous overexpressed RIPK1 and RIPK3 (Fig. 3a, b). In addition to its behavior in dually transfected cells, LMP1 also co-localized with endogenous RIPK1 and RIPK3 when immunofluorescence analysis was performed in CNE1-LMP1 cells (Fig. 3c, d). In our approach, we next performed in situ PLA assay^24^ in 293T cells transiently co-expressing Flag-LMP1 and Myc-RIPK1 or Myc-RIPK3. Consistent with the above results, positive fluorescent signals were observed in the cytosol of cells co-expressing Flag-LMP1 and Myc-RIPK1 or Myc-RIPK3 (Fig. 3e, f), whereas no such signal was observed in cells co-expressing Flag-BHRF1 and Myc-RIPK1 or Myc-RIPK3 (Supplementary Fig. 3), indicating the specific interactions between LMP1 and the two proteins in this cell compartment. Moreover, endougenous RIPK1 and RIPK3 can also interact with LMP1 in EBV-infected cells (Fig. 3g and Supplementary Fig. 4). Overall, these data demonstrate that LMP1 is able to interact directly with both RIPK1 and RIPK3.

### The RHIM-independent interactions rely on LMP1-CTAR2

**Fig. 4:**
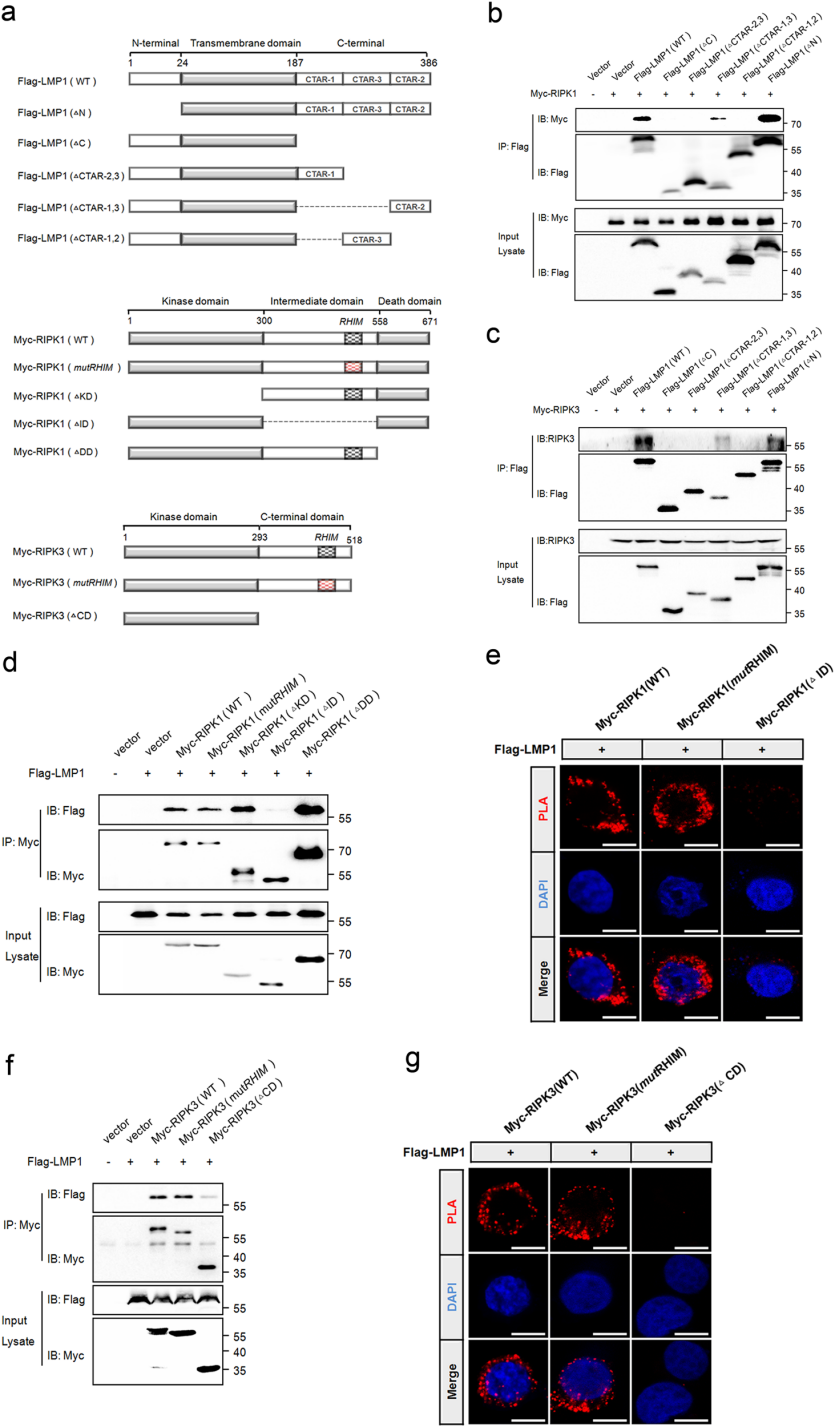
**LMP1-CTAR2 interacts with RIPK1 and RIPK3 in an RHIM-independent manner.** **a** LMP1, RIPK1, and RIPK3 domain architecture and schematic representation of their respective deletion or mutation constructs used in this study. LMP1 (ΔN), the N-terminal domain deletion mutant of LMP1; LMP1 (ΔC), the entire C-terminal domain deletion mutant of LMP1; LMP1 (ΔCTAR-2,3), the CTAR2 and CTAR3 deletion mutant of LMP1; LMP1 (ΔCTAR-1,3), the CTAR1 and CTAR3 deletion mutant of LMP1; LMP1 (ΔCTAR-1,2), the CTAR1 and CTAR2 deletion mutant of LMP1; RIPK1 (*mut*RHIM), the RHIM mutant RIPK1 with AAAA substitutions in IQIG(539-542); RIPK1 (ΔKD), the kinase domain deletion mutant of RIPK1; RIPK1 (ΔDD), the death domain deletion mutant of RIPK1; RIPK1 (ΔID), the entire intermediate domain deletion mutant of RIPK1; RIPK3 (*mut*RHIM), the RHIM mutant RIPK3 with AAAA substitutions in VQVG(458-461); RIPK3 (ΔCD), the C-terminal domain deletion mutant of RIPK3. **b**,** c** IP/IB was used to detect the interaction between Flag-tagged LMP1 mutants and Myc-RIPK1 or Myc-RIPK3. IP of cell lysates employed anti-Flag antibody followed by IB with anti-Myc or anti-Flag-HRP antibody. **d**, **f** IP/IB was used to detect the interaction between Myc-tagged RIPK1 mutants or RIPK3 mutants and Flag-LMP1. IP of cell lysates employed anti-Myc antibody followed by IB with anti-Flag-HRP or anti-Myc antibody. **e**,** g** Proximity ligation assay was used to detect the interaction between Myc-tagged RIPK1 mutants or RIPK3 mutants and Flag-LMP1

LMP1 consists of a N-terminal cytoplasmic domain, six transmembrane domains, and a C-terminal cytoplasmic tail, which contains three C-terminal activating regions (CTARs)^25^. We examined the amino acid sequence of LMP1 in search of an RHIM domain and found none. Therefore, to identify the interaction domain of LMP1 responsible for RIPK1 and RIPK3 binding, we constructed plasmids encoding truncated forms of LMP1 (Fig. 4a). We observed that only the constructs of LMP1 retaining the CTAR2 region were able to bind with RIPK1 as well as RIPK3 in the co-immunoprecipitation assay (Fig. 4b, c), indicating that the CTAR2 region is essential for the interaction between LMP1 and the two receptor-interacting proteins.

The ability of LMP1 to interact with RIPK1 and RIPK3 through its CTAR2 region prompted us to investigate whether the RHIM domain of RIPK1 and RIPK3 is indispensable for this interaction. Because four residues in the RHIM domain of RIPK1 have been shown to be essential for RHIM-based interactions^26^, we generated Myc-tagged RHIM mutant RIPK1 with alanine substitutions in the corresponding four core residues of its RHIM domain, as well as RIPK1 deletion constructs (Fig. 4a). Co-immunoprecipitation experiments showed that LMP1 interacted with RIPK1 (ΔKD) as well as RIPK1 (ΔDD), whereas no interaction was observed between LMP1 and RIPK1 (ΔID), demonstrating the importance of intermediate domain in this interaction (Fig. 4d). Interestingly, the mutation of RHIM within RIPK1 did not abolish the ability of RIPK1 to interact with LMP1. Consistent with the co-immunoprecipitation results, the proximity ligation assay (Fig. 4e) demonstrated an interaction between LMP1 and RIPK1 (*mut*RHIM), but not with RIPK1 (ΔID). Thus, the interaction between RIPK1 and LMP1 occurs independent of its RHIM domain. In addition, we also evaluated the ability of the Myc-tagged RHIM mutant RIPK3, and the RIPK3 deletion construct lacking the C-terminal domain to interact with Flag-LMP1. Similar to the RIPK1 studies, the results of co-immunoprecipitation and PLA also revealed the RHIM-independent interaction between RIPK3 and LMP1 (Fig. 4f, g).

### LMP1 promotes K63-linked polyubiquitination of RIPK1 and suppresses the protein expression

**Fig. 5:**
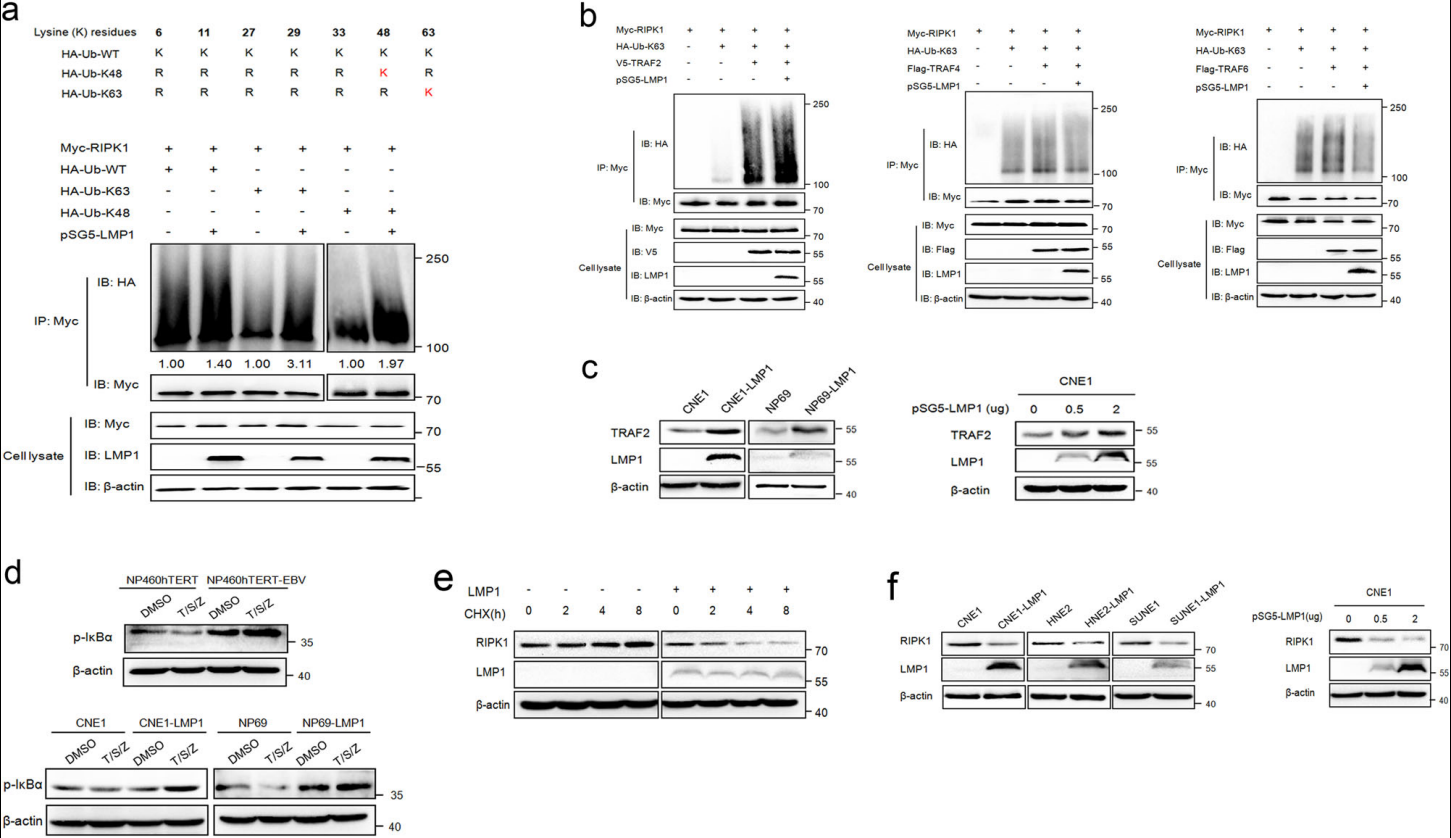
**LMP1 promotes K63-linked polyubiquitination of RIPK1 through TRAF2 and inhibits RIPK1 protein level.** **a** The top panel shows a schematic diagram of ubiquitin mutants. The bottom panel is an in vivo ubiquitination assay performed in 293T cells transfected with vectors encoding Myc-RIPK1, pSG5-LMP1, and HA-UB mutants. Ubiquitinated RIPK1 was detected by IP using anti-Myc antibody followed by IB with anti-HA antibody. The relative levels of RIPK1 ubiquitination were quantified and normalized to vehicle controls. **b** In vivo ubiquitination assay was performed in 293T cells transfected with vectors encoding Myc-RIPK1, HA-Ub-K63, pSG5-LMP1, and V5-TRAF2 or Flag-TRAF4 or Flag-TRAF6 as indicated. K63 ubiquitinated RIPK1 was detected by IP using anti-Myc antibody followed by IB with anti-HA antibody. **c** IB analysis was performed to examine the expression of TRAF2 in CNE1/CNE1-LMP1, NP69/NP69-LMP1 cells as well as LMP1 transiently transfected CNE1 cells. **d** IB analysis was performed to examine p-IκBα expression in NP460hTERT/NP460 hTERT-EBV, CNE1/CNE1-LMP1, and NP69/NP69-LMP1 cells treated with DMSO or T/S/Z. **e** The effect of LMP1 on stability of RIPK1 was examined by cycloheximide chase assay. HNE3 cells transfected with LMP1 or empty vector were treated with cycloheximide (20 μg/ml) for the indicated times. RIPK1 expression levels were determined by immunoblot analysis. **f** IB analysis was performed to examine the expression of RIPK1 in CNE1/ CNE1-LMP1, HNE2/HNE2-LMP1, SUNE1/SUNE1-LMP1 cells as well as LMP1 transiently transfected CNE1 cells

In the TNFα-induced signaling pathway, RIPK1 is a dual-functional signaling molecule that is capable of either pro-survival or pro-death depending on the state of ubiquitination^27^. K63-polyubiquitinated RIPK1 is retained at the plasma membrane, which serves as a docking site to mediate NF-κB activation and thus provides survival signaling, whereas deubiquitination of RIPK1 leads to the formation of complex IIa or complex IIb, initiating apoptosis or necroptosis, respectively^28^. Notably, although LMP1 is not an ubiquitin ligase itself, it serves as an adapter protein for E3 ubiquitin ligases^29,30^ and thus is involved in the regulation of protein ubiquitination^17,31^. We therefore hypothesized that the effect of LMP1 on T/S/Z-induced necroptosis might be mediated by the ubiquitination of RIPK1. To evaluate the impact of EBV and LMP1 on RIPK1 ubiquitination, we performed in vivo ubiquitination assays in EBV-infected cells as well as 293T cells cotransfected with Myc-RIPK1, HA-ubiquitin expression vectors, and LMP1 expression plasmid. Results showed that the levels of RIPK1 ubiquitination in EBV-infected cells was stronger than that of EBV-uninfected cells (Supplementary Fig. 5). Moreover, the existance of LMP1 enhanced the incorporation of wildtype, K63 only and K48 only ubiquitin into RIPK1, indicating the ability of LMP1 to promote these types of polyubiquitin signals (Fig. 5a).

Next, we focused on K63-linked polyubiquitination of RIPK1 and investigated the E3 ligase that might mediate the effect of LMP1 on RIPK1. It has been reported that TNF receptor-associated factor 2 (TRAF2) serves as an E3 ligase for RIPK1^32^. In our study, TRAF2 can interact with and amplify the K63-linked polyubiquitin signal of RIPK1 considerably (Fig. 5b; Supplementary Fig. 6), which was consistent with the reported data. More importantly, addition of LMP1 positively regulated the TRAF2-mediated effect on RIPK1 ubiquitination. We also tested other TRAF proteins TRAF6 (an E3 ligase for NEMO)^33^ and TRAF4 (an E3 ligase for AKT)^34^, but neither was required for this effect (Fig. 5b). Furthermore, immunoblot analysis indicated that LMP1 could upregulate the expression of endogenous TRAF2 (Fig. 5c). Accordingly, LMP1 promotes K63-linked polyubiquitination of RIPK1 through the E3 ubiquitin ligase TRAF2. Upon K63 polyubiquitination, RIPK1 can serve as a scaffold for the recruitment of downstream signaling molecules, which phosphorylate the NF-κB inhibitory protein IκBα and thus result in NF-κB activation^35^. We therefore investigated the effect of LMP1 on NF-κB activity in cells treated with DMSO or T/S/Z. Not surprisingly, the inhibition of IκBα phosphorylation mediated by T/S/Z was prevented in the presence of EBV or LMP1 (Fig. 5d). Taken together, these results support the hypothesis that LMP1 inhibits T/S/Z-induced necroptosis by promoting K63-linked polyubiquitination of RIPK1.

Since K48-linked polyubiquitination mainly targets protein for proteasomal degradation, we therefore performed cycloheximide (CHX) chase assay to determine whether LMP1 affects the degradation of RIPK1 and found that the half-life of RIPK1 was decreased by LMP1. Furthermore, the RIPK1 protein level was also downregulated in response to LMP1 in multiple cell lines (Fig. 5e, f). These data suggested that LMP1 inhibited RIPK1 protein level by shortening its half-life in nasopharyngeal carcinoma cells.

### LMP1 inhibits K63-linked polyubiquitination of RIPK3

**Fig. 6:**
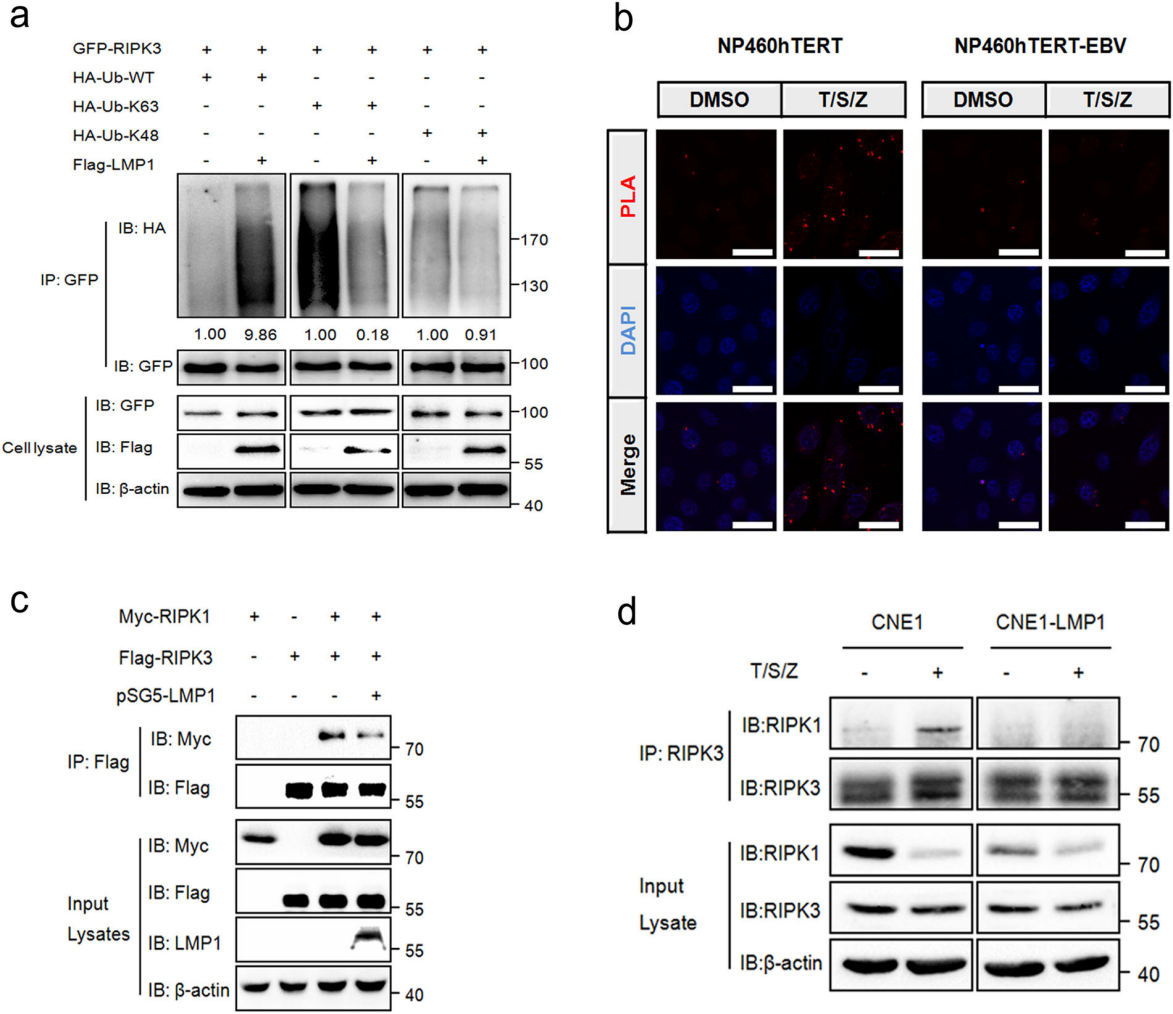
**LMP1 inhibits K63-linked polyubiquitination of RIPK3 and blocks the RIPK1–RIPK3 necrosome formation.** **a** An in vivo ubiquitination assay was performed in 293T cells transfected with vectors encoding GFP-RIPK3, Flag-LMP1, and HA-UB mutants. Ubiquitinated RIPK3 was detected by IP using anti-GFP antibody followed by IB with anti-HA antibody. The relative levels of RIPK3 ubiquitination were quantified and normalized to vehicle controls. **b** Proximity ligation assay was used to detect the RIPK1–RIPK3 interactions in NP460hTERT and NP460hTERT-EBV cells treated with DMSO or T/S/Z. Red fluorescence corresponds to the PLA positive signal and blue fluorescence corresponds to nuclei (DAPI staining). Scale bars represent 25 μm. **c** IP/IB demonstrating LMP1 disruption of the RIPK1 and RIPK3 interaction. Myc-RIPK1, Flag-RIPK3, and pSG5-LMP1 were transfected into 293T cells as indicated. The RIPK1–RIPK3 immunocomplex was analyzed by IP with anti-Flag antibody followed by IB with anti-Myc or anti-Flag-HRP antibody. **d** CNE1 and CNE1-LMP1 cells were treated with DMSO or T/S/Z for 72 h. The RIPK1–RIPK3 immunocomplex was analyzed by IP with anti-RIPK3 antibody followed by IB with anti-RIPK1 or anti-RIPK3 antibody

Although the ubiquitination pattern of RIPK1 has been well characterized, little is known about RIPK3 ubiquitination in the process of necroptosis. Only recently, K63-linked polyubiquitination of RIPK3 has been recognized to be required for the formation of the RIPK1–RIPK3 complex and induction of RIPK3-mediated necroptosis. The deubiquitinating enzyme A20 restricts K63-linked ubiquitination of RIPK3 through its deubiquitinating motif, and thus prevents excessive formation of the necroptotic complex^36^. Interestingly, LMP1 has previously been demonstrated to interact with and upregulate A20^37,38^, which prompted us to investigate whether LMP1 had any effect on RIPK3 ubiquitination or necrosome formation. Our results indicated that co-transfection of LMP1 increased the polyubiqutination of RIPK3 in the setting of ubiquitin wild-type- transfected cells. However, in cells expressing ubiquitin K63, co-transfection of LMP1 remarkably decreased the polyubiqutination of RIPK3 (Fig. 6a). This result suggested that other unknown types of ubiquitination, besides K63 and K48, might probably be regulated by LMP1. Next, RIPK1–RIPK3 interaction was detected by PLA assay in EBV infected and uninfected cells. Results showed that T/S/Z treatment led to enhanced formation of RIPK1–RIPK3 necrosome in NP460hTERT, while this kind of interaction was disrupted in NP460hTERT-EBV (Fig. 6b). In addition, co-immunoprecipitation assay results showed the disruption of the exogenous RIPK1–RIPK3 interaction by LMP1 in 293T cells (Fig. 6c), and IP with RIPK3-specific antibody in CNE1/CNE1-LMP1 cells revealed that LMP1 disrupted T/S/Z-induced endogenous interaction between RIPK1 and RIPK3 to prevent the formation of necrosome (Fig. 6d). Therefore, these results indicate that EBV-LMP1 inhibits K63-linked polyubiquitination of RIPK3, which blocks the RIPK1–RIPK3 necrosome formation.

## Discussion

Cell death is a critical component of the host immune response against invading microbial pathogens. However, the fact that viral species have evolved a variety of mechanisms inhibiting cell death to overcome host defenses against infection is not surprising. So far, viruses equipped to disrupt apoptosis have been extensively studied and well recognized^39^, whereas only a few viral species, such as HSV and CMV, have been analyzed and identified to inhibit necroptosis^11–13^. Therefore, investigating whether other viruses are equipped to block the necroptotic pathway of host cells is interesting. Our present findings provide evidence that another herpesvirus, EBV, is capable of preventing necroptosis. We also demonstrate that EBV-encoded LMP1 contributes to this suppression through a mechanism distinct from either HSV or CMV.

Emerging evidence suggests that the ability of host–pathogen interactions to manipulate host cell death varies among different host species^40^. HSV is the best-known example of this class. Different groups have shown that HSV-1, which is a natural human pathogen but also infects a broad range of animal hosts, has evolved to induce necroptosis in murine cells while blocking this kind of cell death in its natural host, humans^7,8,13^. These seemingly contradictory observations suggest that HSV-1 might have emerged as a human pathogen because of its capacity to block necroptosis specifically in human cells. Unlike its family member HSV, EBV is highly species specific and only infects humans with a restricted cell tropism. Research has long demonstrated the capability of EBV to adapt and evade the host immune defense system^41^. However, whether necroptosis is manipulated by EBV to establish infection and potentiate survival of infected cells is still a mystery. For these reasons, we chose human nasopharyngeal epithelial cells and nasopharyngeal carcinoma cells, which can be infected by EBV, as our cell model to examine the effect of EBV on necroptosis. Our results showed that EBV-uninfected cells were susceptible to T/S/Z-induced necroptosis, but this sensitivity was eliminated by EBV infection, providing direct evidence for virus-specific suppression of the necroptotic pathway.

An important role for the latent viral protein LMP1 in inhibiting necroptosis is suggested by the observation that LMP1-overexpressing cells resisted cell death and showed no alterations characteristic of necroptosis following T/S/Z treatment, while knockdown of LMP1 sensitized EBV-positive cells to this kind of cell death. Previous work has demonstrated the multiple cellular functions of LMP1, including regulation of proliferation, differentiation, and transformation^42–44^. Furthermore, this viral protein also blocks apoptosis by upregulation of anti-apoptotic proteins Bcl-2 and Survivin^15,16^, whereas inhibition of LMP1 enables the generation of an apoptotic complex involving TRADD, FADD, and caspase-8^45^. We have shown here that LMP1 is armed with mechanisms independent of RHIM-domain interactions to block necroptosis. First, LMP1 is able to interact with both RIPK1 and RIPK3. According to the results of PLA, we believe that this interaction is a direct one. Since LMP1 lacks an RHIM, its CTAR2 region is necessary for this RHIM-independent interaction. These findings raise the question of whether LMP1, RIPK1, and RIPK3 exist in a single protein complex. However, this possibility is further excluded by the observation that no RIPK1–RIPK3 complex could be detected in LMP1-overexpressing cells (Fig. 6d). Second, and more importantly, the ubiquitination of the two receptor-interacting proteins can be modulated by LMP1. Unlike K63-polyubiquitinated RIPK1, which serves as a docking site to mediate NF-κB activation and thus provides survival signaling^27^, K63-polyubiquitinated RIPK3 supports the formation of the necrosome and subsequent induction of necroptosis^36^. Our data indicate that LMP1 promotes K63-linked polyubiquitination of RIPK1 and suppresses the protein expression while inhibits K63-linked polyubiquitination of RIPK3 in vivo, collectively switching cell fate from necroptotic death to survival. Accordingly, LMP1-mediated necroptosis inhibition occurs at a step upstream of necrosome formation. Although we found that LMP1 promotes K63-linked polyubiquitination of RIPK1 through the E3 ubiquitin ligase TRAF2, the mechanism of the inhibition of RIPK3 K63-UB by LMP1 was not explored in this study. Ongoing work will further investigate the effect of protein–protein interaction on RIPK1/3 ubiquitination.

Recent studies have demonstrated that necroptosis can be induced by various stimuli, including death ligands (TNFα, FasL, and TRAIL), IFNs, TLR ligands and microbial infection. The initiation of necroptotic response to different stimuli is mediated by different necrotic signaling complexes^46^. The RIPK1–RIPK3 necrosome, which is formed after the ligation of death receptors, characterizes the canonical form of this cell death pathway. Therefore, both RIPK1 and RIPK3 are required for T/S/Z-induced necroptosis^18^. However, other stimuli, such as MCMV, poly(I:C) and IFNβ, can trigger RIPK3-dependent necroptosis completely independent of RIPK1. These “noncanonical” necroptosis pathways are initiated by DAI-RIPK3, TRIF-RIPK3, and another unknown complex^5,11,47^. It should be noted that this study mainly focuses on the effect of EBV on canonical necroptosis induced by T/S/Z and provides detailed evidence that the pathogen-encoded product LMP1 can inhibit necroptosis by regulating RIPK1 as well as RIPK3.

**Fig. 7:**
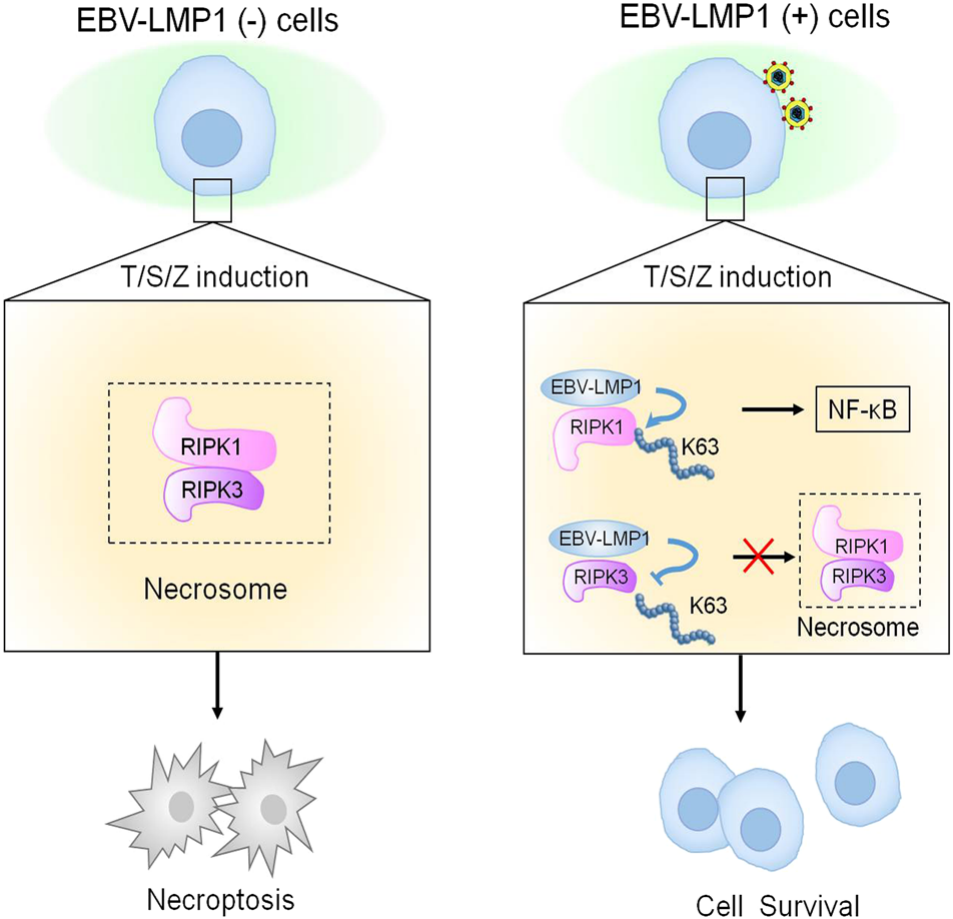
**EBV-LMP1 regulates T/S/Z-induced necroptosis.** EBV-LMP1 (−) cells stimulated with T/S/Z undergo necroptosis through RIPK1–RIPK3 signaling. However, EBV-LMP1 (+) cells can survive under this stimulation. On the one hand, LMP1 interacts directly with both RIPK1 and RIPK3 through its C-terminal activation region. On the one hand, LMP1 promotes K63-linked polyubiquitination of RIPK1 and suppresses the protein expression while inhibiting K63-linked polyubiquitination of RIPK3. These effects contribute to the activation of NF-κB and disruption of necrosome formation, collectively switching cell fate from death to survival

In summary, we demonstrated that the γ-herpesvirus EBV can protect human cells from T/S/Z-induced necroptosis. The latent viral protein LMP1, which has been shown in this study to interact with and modulate the post-translational modification of both RIPK1 and RIPK3, acts as an inhibitor of RIPK1-dependent and RIPK3-dependent programmed necrosis. Based on these findings, we propose a scheme (Fig. 7) to describe LMP1-mediated inhibition of this alternative programmed cell death pathway. The results of the present study provide new insight into the mechanisms of viral manipulation of host cell necroptosis and enrich our knowledge regarding the pathogens armed with strategies to subvert host defenses.

## Materials and Methods

### Reagents and antibodies

Human TNFα and Smac mimetic (GDC-0152) were purchased from Peprotech (Rocky Hill, NJ, USA) and Selleck (Houston, TX, USA), respectively. Necrostatin-1, z-VAD-fmk and CHX were obtained from Sigma-Aldrich (Saint Louis, MO, USA). GSK’872 was from Merck-Millipore (Billerica, MA, USA). Antibodies used were rabbit anti-RIPK1, rabbit anti-Myc, rabbit anti-HA, mouse anti-Myc, rabbit anti-cleaved caspase-3, and rabbit anti-cleaved caspase-8 from Cell Signaling Technology (Danvers, MA, USA), rabbit anti-TRAF2, mouse anti-β-actin, goat anti-rabbit IgG-HRP, and goat anti-mouse IgG-HRP from Santa Cruz Biotechnology (Santa Cruz, CA, USA), rabbit anti-RIPK3, rabbit anti-phosphorylated RIPK3, rabbit anti-phosphorylated MLKL, and mouse anti-phosphorylated IκBα from Abcam (Cambridge, MA, USA), rabbit anti-MLKL, mouse anti-Flag, and mouse anti-Flag-HRP from Sigma-Aldrich, mouse anti-RIPK1 from BD Biosciences (San Jose, CA, USA), mouse anti-LMP1 from DAKO (Glostrup, Denmark), mouse anti-V5 from Invitrogen (Carlsbad, CA, USA), mouse anti-GFP from Thermo Fisher (Waltham, MA, USA).

### Cell culture

The human immortalized nasopharyngeal epithelial cell lines NP460hTERT and NP460hTERT-EBV were grown in 1:1 mixture of Defined Keratinocyte-SFM and EpiLife medium with growth supplements all from Invitrogen. NP69 and NP69-LMP1 were cultured in Defined Keratinocyte-SFM medium. The nasopharyngeal carcinoma cell lines CNE1, CNE1-LMP1, HNE2, HNE2-LMP1, SUNE1, SUNE1-LMP1, C666-1 con, C666-1 shLMP1, and HNE3 were cultured in RPMI-1640 medium from Gibco (Grand Island, NY, USA) supplemented with 10% fetal bovine serum from Biological Industries (Beit Haemek, Israel). The human embryonic kidney cell line HEK293T was cultured in Dulbecco’s modified eagle medium from Gibco supplemented with 10% fetal bovine serum. All cells were cultured at 37 °C in 5% CO2.

### Plasmids and transfections

The expression vectors for Myc-RIPK1, HA-ubiquitin-WT, HA-ubiquitin-K63, HA-ubiquitin-K48, V5-TRAF2, Flag-TRAF4, Flag-TRAF6 and Flag-BHRF1 were from Addgene (Cambridge, MA, USA). The deletion or mutation constructs of LMP1, RIPK1, and RIPK3 were synthesized by Genechem (Shanghai, China), and all of the plasmids were verified by DNA sequencing. Full-length or truncated cDNAs of LMP1 were cloned into BamHI and EcoRI sites of the GV141 vector (CMV-MCS-3FLAG-SV40-Neomycin). Full-length or truncated cDNAs of RIPK1 and RIPK3 were cloned into KpnI and XhoI sites of the GV219 vector (CMV-MCS-SV40-Neomycin) with an N-terminal Myc tag. The *mut*RHIM of RIPK1 and RIPK3 were generated by overlap extension PCR to change RIPK1 aa539–542 from IQIG to AAAA and RIPK3 aa458–461 from VQVG to AAAA. Plasmid transfections were performed using Lipofectamine 2000 from Invitrogen according to the manufacturer’s protocol.

### Cell viability assay and trypan blue exclusion assay

For the viability assay, cells were cultured in 96-well plates. Cell viability was examined using the CellTiter 96 Aqueous One Solution Cell Proliferation Assay from Promega (Madison, WI, USA) according to the manufacturer’s instructions. For trypan blue exclusion assay, cell samples were diluted in Trypan Blue by preparing a 1:1 dilution of the cell suspension using 0.4% Trypan Blue solution from Sigma-Aldrich (Saint Louis, MO, USA). Nonviable cells will be blue, and viable cells will be unstained. The total cells, live cells, or dead cells were automatic counted by Bio-Rad TC10 automated cell counter.

### Cell permeability assay

The cell permeability assay was performed using the cell-impermeable dye Sytox Green from Invitrogen (Carlsbad, CA, USA). Briefly, cells were incubated with 30 nM Sytox Green dead cell stain for 20 min in the dark at room temperature, and then visualized with a Leica DMI3000 fluorescence microscope.

### Immunoblotting and immunoprecipitation

Cells were harvested using Pierce IP lysis buffer from Thermo Fisher supplemented with complete protease inhibitor cocktail from Roche (Basel, Switzerland). For immunoblotting, cell lysates were subjected to SDS-PAGE and blotted with appropriate primary antibodies as well as secondary antibodies labeled with horseradish peroxidase. Visualization of proteins was performed using the ChemiDoc XRS system with Image Lab software (Bio-Rad). For immunoprecipitation, cell lysates were incubated with specific antibody followed by incubation of protein G beads from Life Technologies (Carlsbad, CA, USA). The precipitated proteins were eluted from resuspending the beads before immunoblotting.

### Confocal microscopy and transmission electron microscopy

For confocal microscopy, cells were fixed in 4% paraformaldehyde and incubated in blocking buffer containing 5% donkey serum and 1% bovine serum albumin. After incubation with rabbit anti-RIPK1 or anti-RIPK3 and mouse anti-LMP1 antibodies, Alexa Fluor 594-conjugated anti-rabbit IgG (A-21207) and Alexa Fluor 488-conjugated anti-mouse IgG (A-11001) purchased from Thermo Fisher were added, respectively. The cells were incubated with DAPI from Sigma-Aldrich to stain nuclei. Images were acquired on a Leica TCS SP5 confocal microscope. For TEM, cells were fixed in 2.5% glutaraldehyde in 0.1 M cacodylate buffer (pH 7.2). The cells were washed in 0.1 M cacodylate buffer, then fixed with the same buffer with 1% osmium tetroxide, washed, and dehydrated through a graded series of ethanol to 100% and embedded in epoxy resin. The sections were counterstained with uranyl acetate and lead citrate. Images were acquired on a Hitachi HT7700 TEM operated at an accelerating voltage of 100 kV.

### Proximity ligation assay

The DuoLink^®^ In Situ Red Starter Kit Mouse/Rabbit (DUO92101) purchased from Sigma-Aldrich was used for this experiment. Cells seeded in eight-well chamber slides were washed with PBS, fixed in 4% paraformaldehyde for 30 min, and then permeabilized in 0.1% Triton X-100 for 20 min. Slides were then blocked with Duolink blocking solution in a pre-heated humidity chamber for 30 min at 37 °C and incubated with the primary antibody to detect Myc/Flag (or RIPK1/RIPK3) overnight at 4 °C. On the following day slides were incubated with the PLA probes diluted 1:5 in antibody diluents in a pre-heated humidity chamber for 1 h at 37 °C. Subsequent hybridization, ligation, amplification, and detection were performed according to the manufacturer’s protocol. Fluorescence images were acquired using a Leica TCS SP5 confocal microscope.

### In vivo ubiquitination assay

Ubiquitination assays were performed as described^48^. For in vivo ubiquitination assay of RIPK1, 293T cells were transfected with Myc-RIPK1, HA-Ub (WT), or HA-Ub mutants and pSG5-LMP1 for 48 h. Cell lysates were heated at 95 °C for 10 min in 1% SDS to dissociate proteins and diluted 10 times in buffer without SDS. Myc-RIPK1 was immunoprecipitated using anti-Myc antibody at 4 °C overnight before incubation with protein G beads. Samples were washed and prepared for immunoblot analysis as described above.

## Electronic supplementary material

Supplementary Table 1

Supplementary Table 2

Supplementary figure 1

Supplementary figure 2

Supplementary figure 3

Supplementary figure 4

Supplementary figure 5

Supplementary figure 6

Supplementary figure legends
