# Supplementary material for: Epstein-Barr virus encoded latent membrane protein 1 suppresses necroptosis through targeting RIPK1/3 ubiquitination
Source: Cell Death Dis. 2018 Jan 19;9(2):53. doi: 10.1038/s41419-017-0081-9 (PMC5833833; doi:10.1038/s41419-017-0081-9)
Supplement: Supplementary file 2 — Supplementary Table 2 [file 41419_2017_81_MOESM2_ESM.docx]

**Table S2. The average percentage of necrosis cells (necrosis rate) calculated by TEM image analysis in CNE1 and CNE1-LMP1 cells.**

| Treatment | CNE1 | CNE1-LMP1 |
| --- | --- | --- |
|  | Necrosis rate (over 200 cells counted) | |
| DMSO | 1.8% | 1.9% |
| T/S/Z | 10.2% | 2.3% |
