# Supplementary figures and images for: Epstein-Barr virus encoded latent membrane protein 1 suppresses necroptosis through targeting RIPK1/3 ubiquitination
Source: Cell Death Dis. 2018 Jan 19;9(2):53. doi: 10.1038/s41419-017-0081-9 (PMC5833833; doi:10.1038/s41419-017-0081-9)

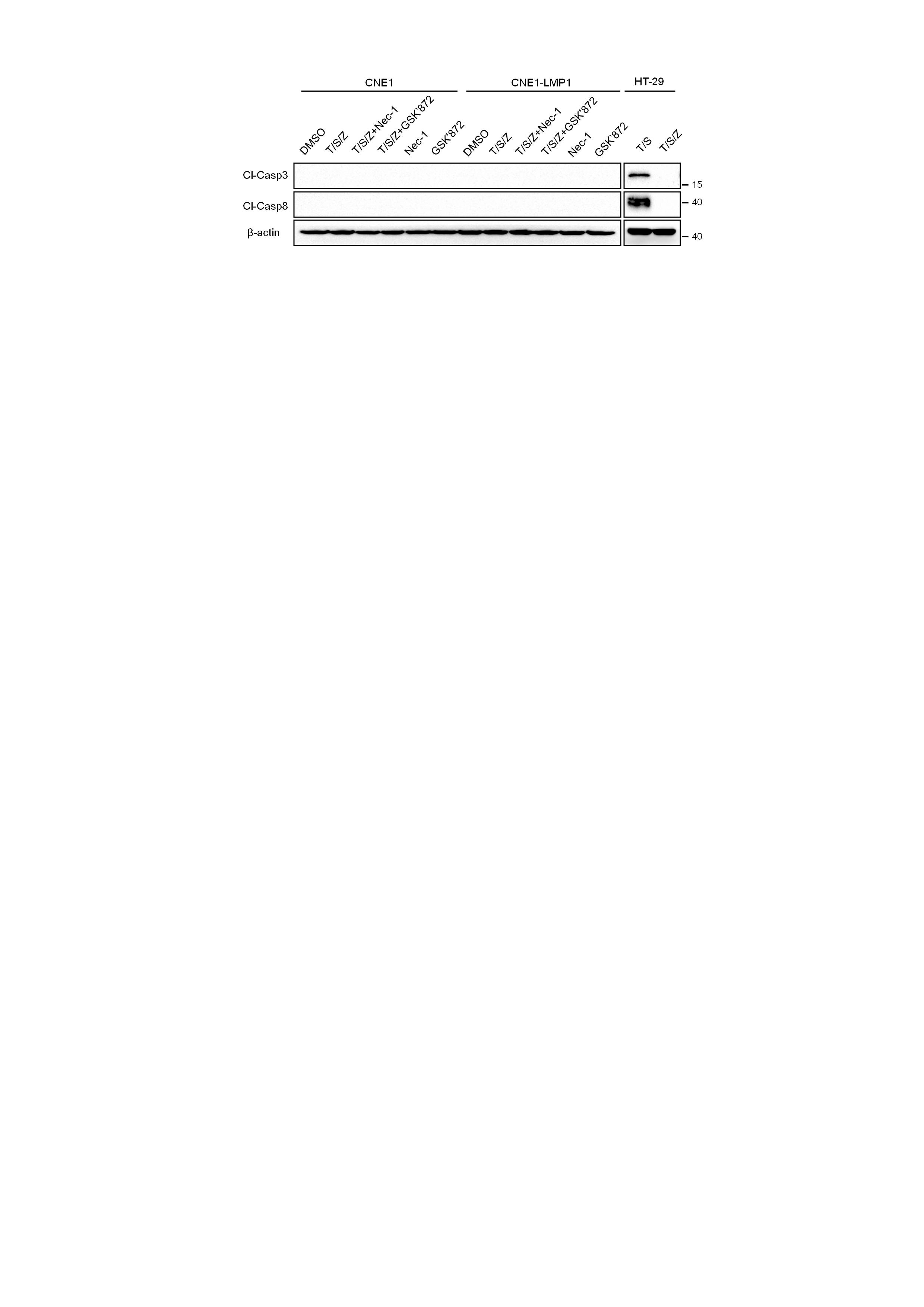

Supplement: Supplementary file 3 — Supplementary figure 1 [file 41419_2017_81_MOESM3_ESM.tif]

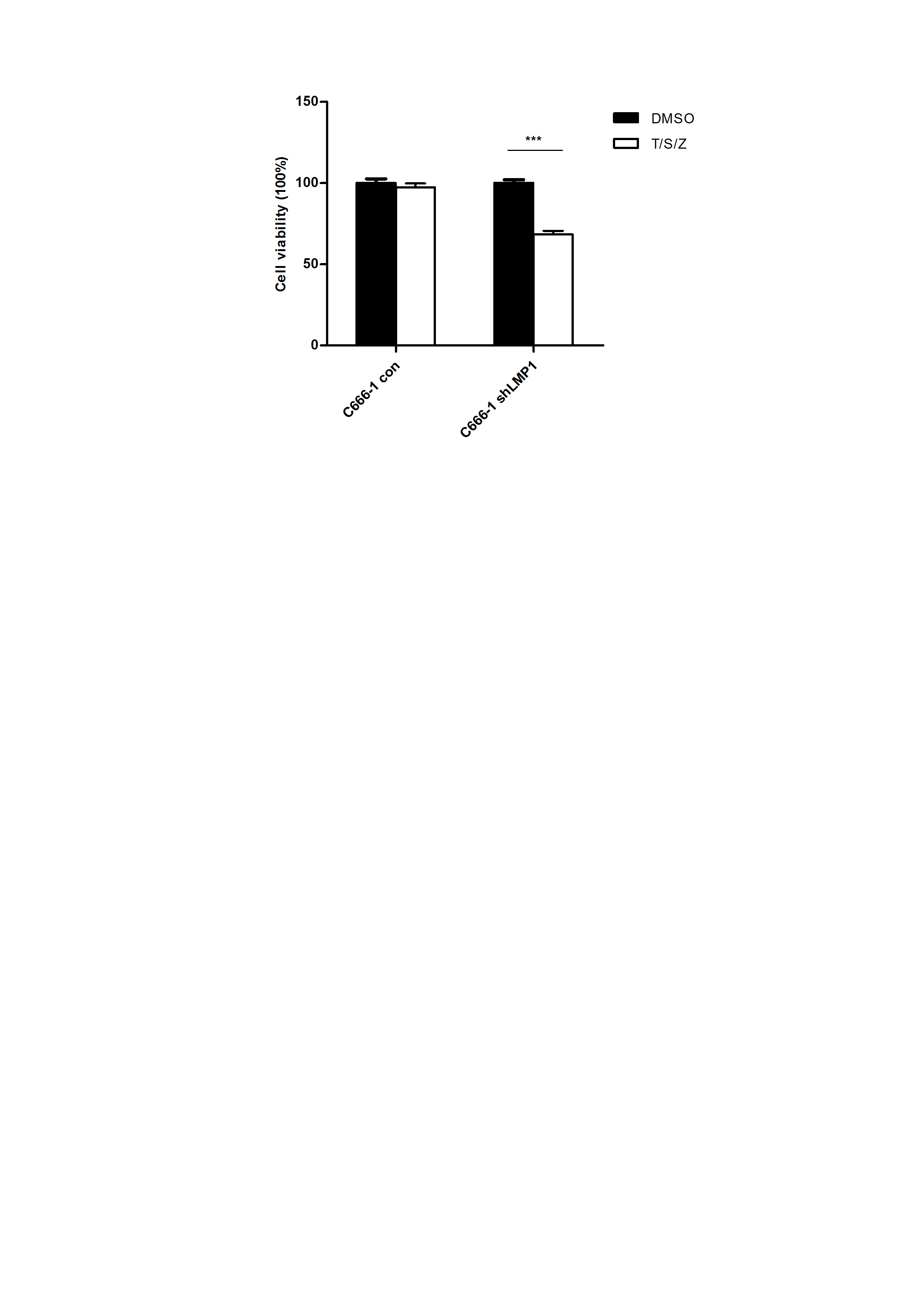

Supplement: Supplementary file 4 — Supplementary figure 2 [file 41419_2017_81_MOESM4_ESM.tif]

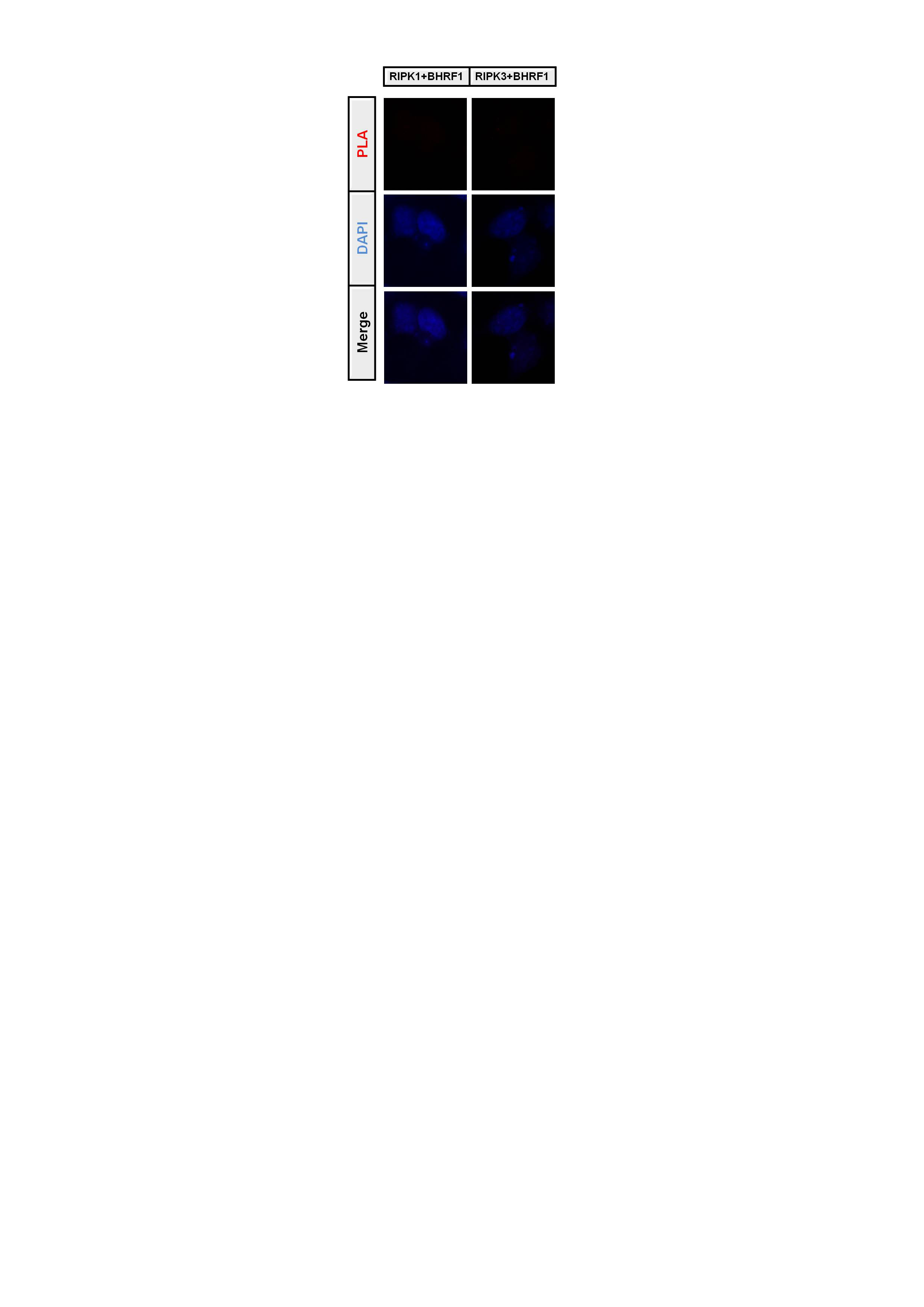

Supplement: Supplementary file 5 — Supplementary figure 3 [file 41419_2017_81_MOESM5_ESM.tif]

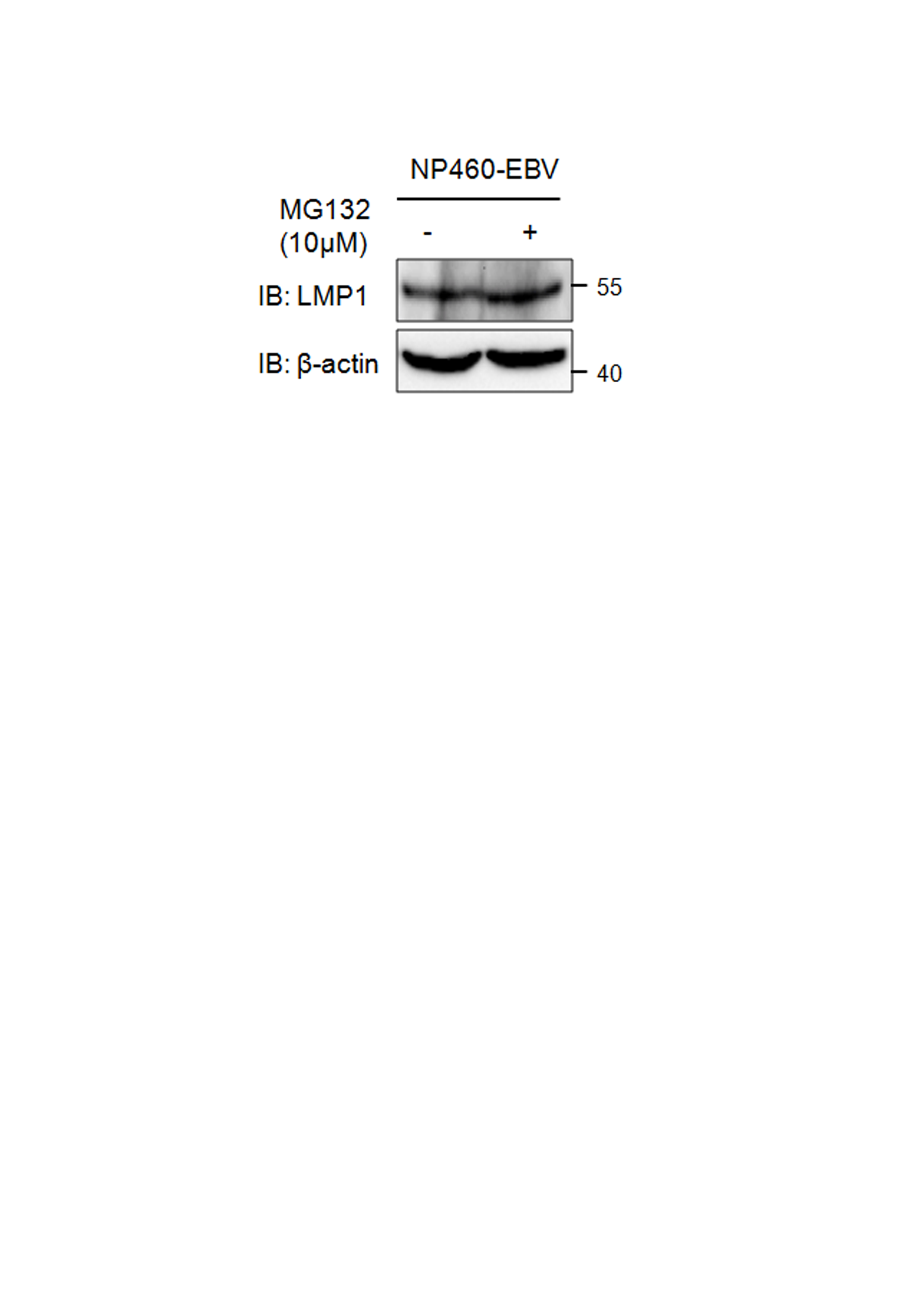

Supplement: Supplementary file 6 — Supplementary figure 4 [file 41419_2017_81_MOESM6_ESM.tif]

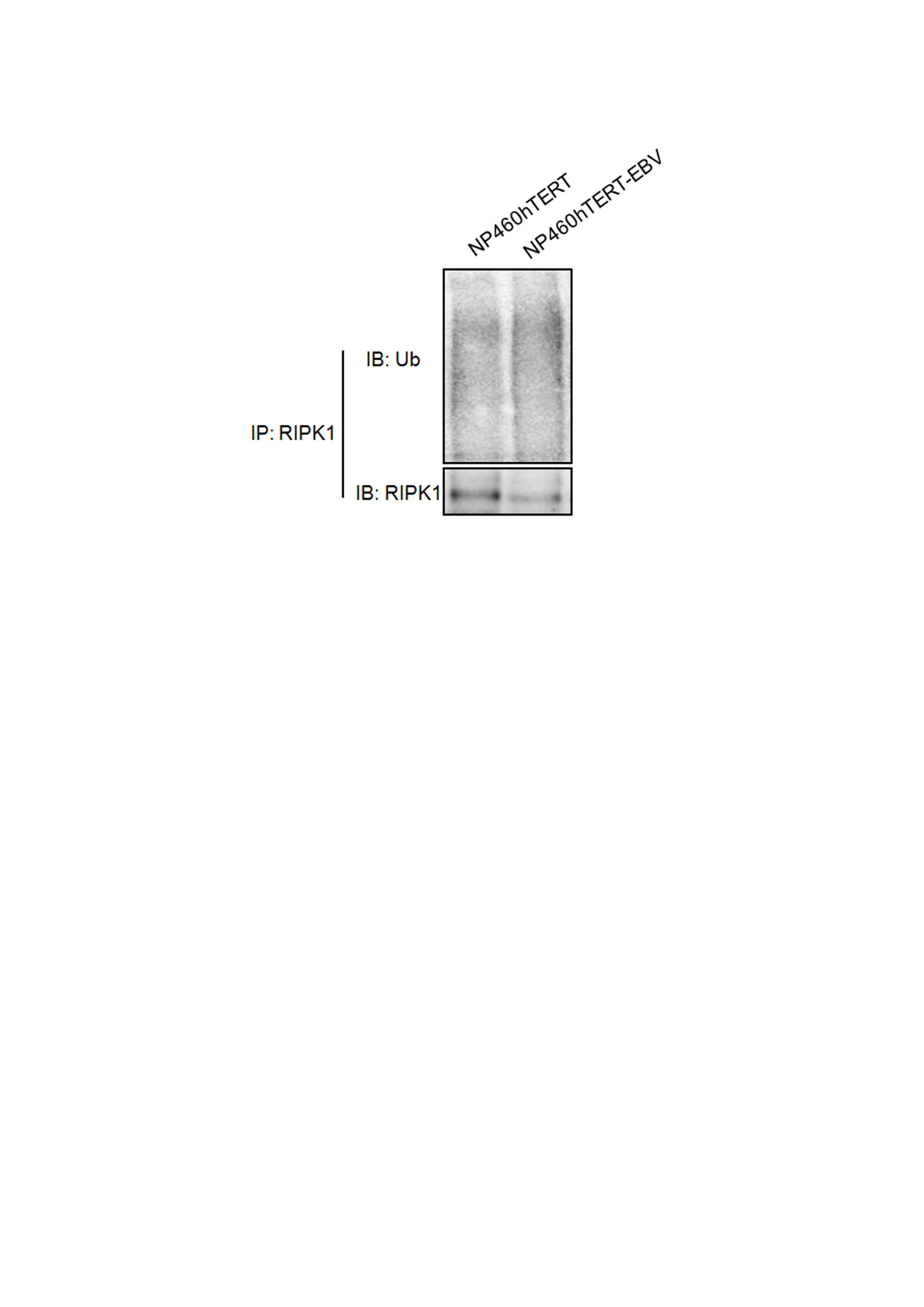

Supplement: Supplementary file 7 — Supplementary figure 5 [file 41419_2017_81_MOESM7_ESM.tif]

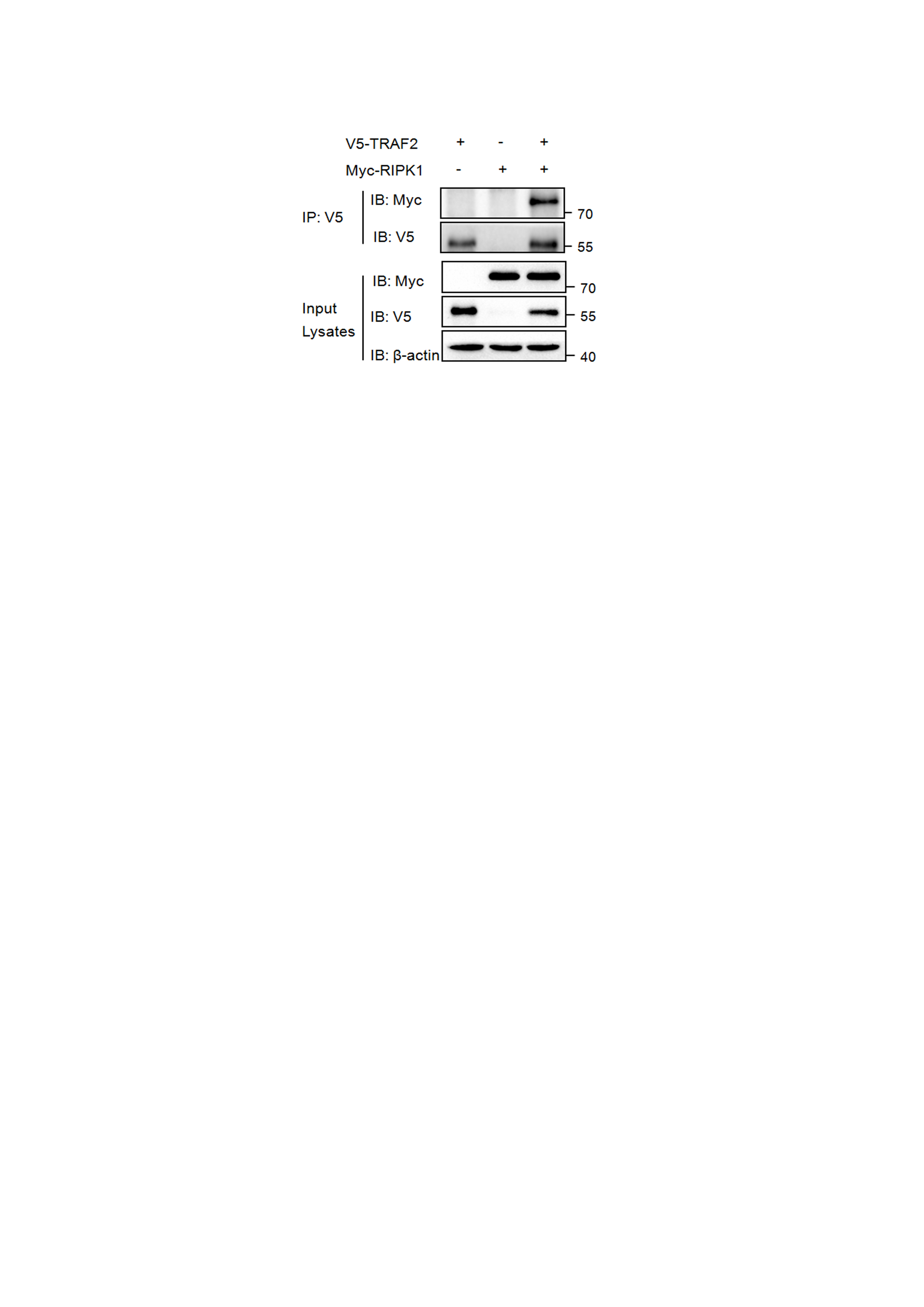

Supplement: Supplementary file 8 — Supplementary figure 6 [file 41419_2017_81_MOESM8_ESM.tif]
