# Supplementary material for: Epstein-Barr virus encoded latent membrane protein 1 suppresses necroptosis through targeting RIPK1/3 ubiquitination
Source: Cell Death Dis. 2018 Jan 19;9(2):53. doi: 10.1038/s41419-017-0081-9 (PMC5833833; doi:10.1038/s41419-017-0081-9)
Supplement: Supplementary file 9 — Supplementary figure legends [file 41419_2017_81_MOESM9_ESM.docx]

**Supplementary figure legends**

**Figure S1. The type of cell death in CNE1 is not apoptosis.** Immunoblot analysis to detect cleaved Caspase-3 (Cl-Casp3), cleaved Caspase-8 (Cl-Casp8) and β-actin in lysates from CNE1 and CNE1-LMP1 cells treated with T/S/Z, T/S/Z+Nec-1, T/S/Z+ GSK’872, Nec-1, GSK’872 or DMSO solvent control and HT-29 cells treated with T/S or T/S/Z as indicated.

**Figure S2. Knockdown of LMP1 sensitizes EBV-positive cells to T/S/Z induced necroptosis.** C666-1 con and C666-1 shLMP1 cells were treated with T/S/Z or DMSO solvent control for 48 hours. At the end of treatment, cell viability was determined by MTS assay.

**Figure S3. BHRF1 does not interact with RIPK1 or RIPK3.** Proximity ligation assay was used to detect the BHRF1-RIPK1 and BHRF1-RIPK3 interactions in 293T cells transfected with the indicated expression plasmids (top of each panel). Red fluorescence corresponds to the PLA positive signal and blue fluorescence corresponds to nuclei (DAPI staining).

**Figure S4. MG132 stabilized LMP1 expression.** NP460hTERT-EBV cells were treated with 10μM MG132 or DMSO solvent control for 16 hours. LMP1 expression levels were determined by immunoblot analysis.

**Figure S5. RIPK1 ubiquitination in EBV-infected cells was stronger than that of EBV-uninfected cells.** An *in vivo* ubiquitination assay was performed in NP460hTERT and NP460hTERT-EBV cells. Ubiquitinated RIPK1 was detected by IP using anti-RIPK1 antibody followed by IB with anti-Ub antibody.

**Figure S6. The E3** [**ubiquitin**](http://www.nature.com/nature/journal/v465/n7301/abs/nature09128.html) **ligase TRAF2 interacts with RIPK1.** IP/IB was used to detect the interaction of TRAF2 with RIPK1. Top panels show lysates of 293T cells transfected with V5-TRAF2 and/or Myc-RIPK1 subjected to IP with anti-V5 antibody followed by IB with anti-Myc or anti-V5 antibody. The lower panels depict IB of input cell lysates. Protein molecular size is shown to the right of the lanes.
